# Medical News and Bulletin

**Published:** 1843-08-05

**Authors:** 


### NEW YORK JOURNAL OF MEDICINE.

We have received the first number of "the New York Journal of Medicine and the Collateral Sciences," edited by Samuel Forry, M. D., for July, 1843; and published by the Messrs. Langley, 57 Chatham street, at three dollars per annum in advance. The first number impresses us very favourably, and when we consider the ample field New York presents, both among its Hospital and private practitioners, for the support of a Medical Journal, united to the abilities of the editor, we do not doubt but that the succeeding numbers will be still more interesting and able.

---

Dumoulin, a celebrated French practitioner of former times, never lost sight of the fact that the payment of the fee is an essential part of the visit to a patient. If asked, "Shall you come again, doctor? he would reply, "Yes, if you pay me." "Must I pay you now?" "Yes, if you wish me to come again!" Having, with a brother-physician been called to attend a nobleman who was dangerously ill, both doctors arrived at the house one day when the patient was *in articulo mortis*. The servants in the hall were full of invectives, and audibly threatened the disciples of Æsculapius with summary punishment in case their master should die. This catastrophe having taken place, Dumoulin's colleague, terrified at the impending penalties, anxiously inquired of him by what door they should get out. "By the door they pay at," said Dumoulin, and followed by the other, he boldly traversed the hall, and went to demand his fee.—*Lond. Lancet.*

---

### HOW TO MAKE LEECHES BITE.

The leech which it is intended to apply is to be thrown into a saucer containing fresh beer, and is to be left there till it begins to be quite lively. When it has moved about in the vessel for a few moments, it is to be quickly taken out and applied. This method will rarely disappoint expectation, and even dull leeches, and those which have been used not long before, will do their duty. It will be seen with astonishment how quickly they bite.—*Lond. Med. Gaz., from Weitenweber's Beitr., and Schmidt's Jahrb.*

---

### CAT'S MINT.

The *Nepeta cataria* of Linnæus is recommended by Dr. Guastamacchia as a sovereign remedy for tooth-ache, whether it proceeds from catching cold, or from caries. The leaves of the plant are placed between the affected tooth and the opposite one; this causes a copious flow of saliva, and in two or three minutes the most violent pains are relieved. If the patients cannot keep the leaves in contact with the diseased tooth, they must chew them, and the object is equally attained by the flow of saliva thus excited.—*Ibid, from Filiatre Sebezio, and Schmidt's Jahrb.*

### A GLUT OF DOCTORS.

It appears that the profession is overstocked with practitioners in France as well as in England: more than forty French doctors in medicine having sometime since applied to the Government for free passage to the Marquesas Islands.—*Lon. Lancet.*

---

### DISEASES IN ITALY.

According to a new Belgian medical traveller, Italy, the garden of Europe, south of the Alps and Apennines, is a very unfit region for individuals suffering from pectoral diseases. At the hospital of Pammatone, in Genoa, of a number of patients received during a period not stated previously to Jan. 1833, 2354 were the subjects of chest disease; while only 735 were affected with abdominal diseases, 460 with inflammatory fevers, 59 with intermittent fevers, and 529 with syphilitic diseases. Tubercular disease is very common at Florence, Naples, and Rome; and at the last named city the deaths annually are as 1 to 26 of the inhabitants (Tournon *Statist. de Rome;*) while in the country at large it is but as 1 to 23. Independently of the prevalence of malaria in its neighbourhood, Rome, in common with most Italian cities near the sea, experiences great variations of temperature. Calculi and hepatic and cerebral diseases are very common in Italy. Insanity is, however, much less common than in England, for at the time that in our own country, with 12,780,000 inhabitants, there were estimated to be upwards of 17,200 insane persons, Italy, with 16,799,000 inhabitants, was supposed to comprise only 1500 insane. *Ibid.*

---

### FREQUENCY OF SECOND ATTACKS OF VARIOLA.

From observations made in 1,500 cases of small-pox, M. Serres has arrived at the conclusion that secondary attacks of that disease are as common after variola itself as after vaccination, and he believes that vaccination has simply the effect of preventing a first attack of small-pox, being merely of the same efficacy in that respect as an attack of the disease itself.—*Provincial Medical Journal.* June 24, 1843.

---

### DECOCTION OF OAK-BARK.

This preparation has of late been strongly recommended by a French practitioner as an injection into dropsical cysts, as hydrocele, &c., after their previous contents have been drawn off. It is said to exert a marked tendency in preventing a subsequent accumulation of fluid. Its active astringent quality suggested to the above practitioner that it might be serviceable in promoting the contraction of the ring after the reduction of recent inguinal hernia; and the application for some time of compresses impregnated with a strong decoction of oak-bark, kept *in situ* by a truss or bandage, has been in his practice attended with this result to the most satisfactory extent.—*Lon. Lancet, from Gaz. Med.*

